# Annotations

**Published:** 1888-10-27

**Authors:** 


### ANNOTATIONS.

**Lady Guardians.**

WHAT wondrously wise creatures men are, or think they are! According to Miss Louisa Twining there are only two lady poor law guardians in all England north of the Trent. In Scotland and the South of England the proportion is slightly greater, but still ridiculously small. One would really think, to hear men talk, that nothing ever went wrong which was fortunate enough to be placed under their guidance and control. As a matter of fact, however, the things that go absolutely right as the result of being guided solely by committees of men do not exist. Every man who has had experience of any kind of public work knows that the rule is this, that the work does somehow or other get done in some kind of fashion, and that what committees accomplish is to see that blundering and stupidity and inefficiency are kept in some kind of check. Committees, boards of guardians, and other public bodies of the like class are, very frequently, mere vendors of more or less twaddle. Here and there a man of real capacity may be found; but he often finds it so trying to his temper to sit and listen to the rubbish which his fellow committeemen will gravely propose, that he not seldom ends by refusing to act on committees at all. We do not say that all this would be mended by electing ladies to boards of guardians. What we do say is that ladies could by no means add to the stupidity of many of the boards which already exist, whilst they might possibly contribute something in the way of simplicity and liveliness. But in truth it seems necessary that the woman element should always make itself felt where women and children are concerned. The majority of paupers are women and children. They cannot but frequently need that some kind of womanly supervision of their circumstances shall be made. It is possible to imagine, even among paupers, tender and shrinking women and sensitive and nervous children, who suffer much at the hands of cold hearted and dull-brained officials. Men guardians are slow to find faults which lie below the surface and in the region of the sentiments. Women would go down deep into such questions at once. Not only do we deeply sympathise with the movement which aims at increasing the number of lady guardians, but we express the emphatic conviction that no guardians' board should be considered properly constituted which does not include at least one or two women in its number.

**Murder and "Penny Dreadfuls."**

THE confession of murder by the two lads, Gower and Dobell, at Tunbridge Wells, calls attention once more to the kind of literature which is supplied to and read by boys and girls of the artisan class. Here are two mere youths, little more than children, who coolly and deliberately plan a murder, and as coolly and deliberately execute it. In due time they confess what they have done, and add to their confession a number of other details of crime, which show them to have been entirely possessed and governed by ideas of robbery, murder, and other kinds of villainy. The whole thing is done in cold blood, and confessed in cold blood. There is no question of momentary impulse, or sudden yielding to sudden temptation. The mind of each of the youths was evidently prepared for a life of the wild beast kind, in which conscience and morals play no part at all. It is the interest of the public, to say nothing of duty, to inquire into the previous history of these two youths. There is plenty of time to do so. What is their family history? What influences of heredity can be discovered? Perhaps none! It may be that the parents of the lads are of the well-doing and moral working class. But if heredity has had nothing to do with such abnormal developments, what part has environment played? Where were the early school-days of these children spent? What kind of man was their school-master? Above all, what influences have they been subjected to since their school-days were left behind? It is stated that a variety of wicked and filthy books were discovered in their homes. If that be so it ought to be made known far and wide what these books were. Perhaps well-directed questions might evoke answers which would throw full light on the whole question of youthful depravation by means of immoral literature. These lads should not be merely destroyed like noxious animals; they should be used for moral and psychological purposes. If they proceeded to the extremity of deliberate murder in cold blood, who knows how many hundreds and thousands of lads in Tunbridge Wells and elsewhere, whilst stopping short of that, may yet be so entirely demoralised as to be dangerous criminals in purpose and intent, only waiting for the temptation and the opportunity? The question of depraving literature demands thorough inquiry. Of course, it bristles with difficulties; but so does the subject of food and drug adullteration. If it be dangerous to poison the body, it is much more dangerous, from a statesman's point of view, to poison the mind. It would be quite possible to find out generally the depth and extent to which immoral literature is affecting the character of the people, and with the energy of public opinion which we have in favour of virtue in this country, it ought not to be impossible to deal effectually with the subject.

**The Tyrannies of the Workshop.**

EVERY man is more or less under the influence of his calling. The lawyer usually has the worst possible opinion of human nature because he so often sees its worst side, and so seldom its best. The man of business is so accustomed to act on what he calls "strict business principles" that he comes to look on the world as a great mart, and a mart only. The doctor, on the other hand, spends his days in striving to allay the sufferings and to repair the damage inflicted in the battle of life, and he looks upon the world as a huge hospital, and often a very sad one. If he could, he would moderate many hardships and lessen many severities inflicted upon the poor, the defenceless, and especially the young. Our contemporary, *The Star*, calls attention to the system of "fining" workmen, women, and boys, which, in spite of its illegality, is still practised at many business establishments. Two cases are recorded, one that of a boy of thirteen, who, being required to work from six a.m. to six p.m. by the rules of his firm, was kept at home by his mother, so as to shorten the hours considerably. Therein the mother showed herself a sensible and feeling woman. The wages of the boy were of course diminished in strict proportion to his shortened hours, and against that we have nothing to say. But, in addition, fines were imposed amounting to rather more than 3*d*. a-day, or 1*s*. 4*d*. for five days. As the total earned by the boy amounted to 3*s*. 8½*d*., the deduction of 1*s*. 4*d*. for fines left him only 2*s*. 4½*d*. for nearly a week's work of ten hours daily, or less than 6*d*. a-day. Now we readily admit that discipline must be maintained in business establishments; and probably the heads of firms would say that fines are the shortest and easiest methods of discipline. But everything cannot be sacrificed to short and easy methods, or to the convenience of employers. Reverse the case: put the boot on the other leg. Suppose that instead of the boy transgressing against the master, the master had transgressed against the boy—what short and easy method of punishment could the boy have adopted? Absolutely none! He would have had to seek his remedy either in leaving the service or in the law courts. The master also should be compelled to do the same thing. Is it objected that that would be both inconvenient and costly to the master? True, but it would be relatively ten times more inconvenient and costly for the boy. "What is sauce for the goose is emphatically sauce for the gander," unless, indeed, the gander be so old and tough as not to be worth either sauce or cooking. This boy had committed absolutely no fault. His mother wished him to work from eight a.m to six p.m. only. It clearly suited the employers to have him on those conditions, because they still continued to employ him. Their fines were illegal, and as irrational and unjustifiable as they were illegal. The boy was thirteen, and therefore, perhaps, past the school age; but does the Factory Act permit such children to work from six to six? Questions of this kind demand the attention of working men's associations. Very properly, workmen, as a class, have long since awakened to the necessity of mutual association for mutual protection. Trade societies should make a point of putting down the fine system, and of compelling employers to seek redress by the same constitutional methods which the employed are obliged to use.

